# Progressive Fibrosing Lung Disease Treated With Nintedanib in a Patient With Long‐Standing Autoimmune Pulmonary Alveolar Proteinosis: A Case Report

**DOI:** 10.1155/crpu/8073947

**Published:** 2026-07-31

**Authors:** Yoshiaki Zaizen, Yasuhiko Nikaido, Takeo Jimi, Tetsuya Kawano, Katsuyuki Ichiki, Toru Tsuda, Tomoaki Hoshino

**Affiliations:** ^1^ Division of Respirology, Neurology and Rheumatology, Department of Medicine, Kurume University School of Medicine, Kurume, Fukuoka, Japan, kurume-u.ac.jp; ^2^ Department of Respiratory Medicine, Kirigaoka Tsuda Hospital, Kitakyushu, Fukuoka, Japan

**Keywords:** antifibrotic agent, autoimmune pulmonary alveolar proteinosis, interstitial lung disease, nintedanib, progressive pulmonary fibrosis

## Abstract

**Background:**

Autoimmune pulmonary alveolar proteinosis (APAP) is caused by impaired surfactant clearance due to neutralizing autoantibodies against granulocyte‐macrophage colony‐stimulating factor. Although whole‐lung lavage and inhaled granulocyte‐macrophage colony‐stimulating factor therapy are established treatment options, pulmonary fibrosis is increasingly recognized as a clinically relevant complication in a subset of patients with APAP. However, the clinical behavior of APAP‐associated fibrosing lung disease and the role of antifibrotic therapy remain unclear.

**Case Presentation:**

A 54‐year‐old man with a 15‐year history of APAP was referred to our institution. High‐resolution computed tomography images obtained before referral showed slow progression of reticulation and traction bronchiectasis, suggesting fibrotic progression rather than recurrence of APAP. At presentation, forced vital capacity (FVC) was 3.31 L (80.0% predicted), and diffusion capacity for carbon monoxide (DL_CO_) was preserved. During 6 months of observation, FVC declined to 3.03 L (73.5% predicted), accompanied by worsening dry cough and exertional dyspnea. Nintedanib was initiated for a progressive fibrosing phenotype in the context of APAP. Thereafter, FVC remained relatively stable for 2 years, whereas DL_CO_ declined during follow‐up.

**Conclusions:**

APAP‐associated fibrosing lung disease may present with a progressive fibrosing phenotype, but its diagnosis and management remain challenging. This case highlights the importance of distinguishing fibrotic progression from recurrence of intra‐alveolar proteinosis.

## 1. Introduction

Pulmonary alveolar proteinosis (PAP) is a rare disorder characterized by abnormal accumulation of eosinophilic, granular, and surfactant‐derived materials within the alveolar spaces due to impaired surfactant production or clearance [1]. Autoimmune pulmonary alveolar proteinosis (APAP) is caused by neutralizing autoantibodies against granulocyte‐macrophage colony‐stimulating factor (GM‐CSF), leading to functional impairment of alveolar macrophages and neutrophils [1]. Whole‐lung lavage (WLL) is a standard treatment for symptomatic APAP. Although WLL often provides disease control, some patients relapse and require repeated lavage.

Recently, inhaled recombinant human GM‐CSF therapies, including sargramostim and molgramostim, have emerged as therapeutic options for APAP and have demonstrated favorable clinical outcomes [1]. However, pulmonary fibrosis has increasingly been recognized as a clinically relevant complication in a subset of patients with APAP. Current guidelines define progressive pulmonary fibrosis (PPF) as a progressive fibrosing phenotype occurring in interstitial lung diseases other than idiopathic pulmonary fibrosis [2], and antifibrotic agents have demonstrated efficacy in reducing forced vital capacity (FVC) decline in patients with PPF [3, 4]. However, APAP‐associated fibrosing lung disease remains uncommon in clinical practice, and evaluating disease progression and treatment response can be challenging.

Herein, we report a patient with a 15‐year history of APAP who developed a progressive fibrosing phenotype and was treated with nintedanib during the clinical course. This case illustrates the diagnostic and therapeutic challenges of fibrosing lung disease in patients with APAP.

## 2. Case Presentation

A 54‐year‐old man was referred to our institution for continued management of APAP after discontinuing follow‐up at a regional general hospital. The patient had been evaluated 15 years earlier for respiratory failure and was subsequently diagnosed with APAP. At that time, serum and bronchoalveolar lavage fluid showed markedly elevated anti‐GM‐CSF antibody. Over the following 2 years, he underwent three sessions of WLL during three admissions. His disease then stabilized, and he was referred to a regional general hospital for follow‐up 13 years before the current presentation. He experienced no recurrence of respiratory failure during this period, and CT and pulmonary function tests were not routinely performed. As continued follow‐up at the general hospital was deemed unnecessary, he was referred to our institution.

At the first visit to our institution, his resting percutaneous oxygen saturation was 97% in room air; however, he reported dyspnea on exertion corresponding to Grade 1 on the modified Medical Research Council dyspnea scale. This symptom had not been reported at his last visit to the referring institution 1 year earlier. Arterial blood gas analysis in room air showed a PaO_2_ of 68 Torr, corresponding to disease severity score (DSS) grade 3 for APAP. He walked 470 m in the 6‐min walk test, and the lowest SpO_2_ during exertion was 92% in room air. The Krebs von den Lungen‐6 level was elevated at 3180 U/mL. Anti‐GM‐CSF antibody was not reassessed at the time of referral to our institution.

**Figure 1:**
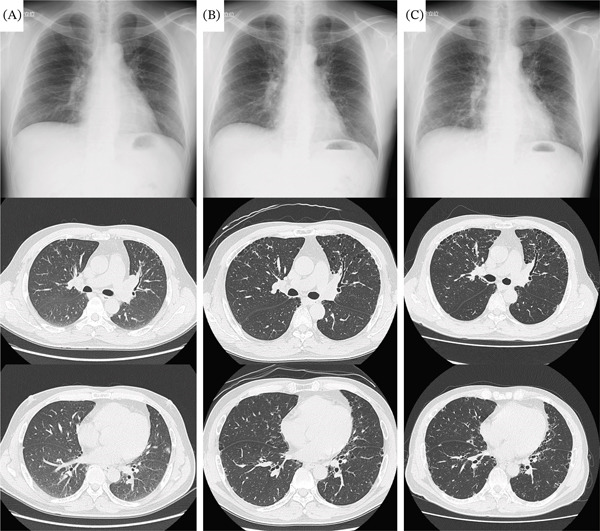
Comparison of chest radiography and high‐resolution computed tomography (HRCT) imaging prior to referral to our hospital. Progression of linear opacities, reticulation and traction bronchiectasis is evident. (A) Thirteen years before referral—2 years after the patient underwent whole lung lavage procedures. (B) Two years before referral. (C) Four months before referral.

The oldest available chest high‐resolution computed tomography (HRCT) images had been obtained 13 years before referral, 2 years after the patient underwent WLL. Serial HRCT images obtained 13 years, 2 years and 4 months, and 4 months before referral showed gradual progression of reticulation and traction bronchiectasis (Figure 1). The predominant radiological changes were fibrotic abnormalities rather than newly developed or worsening ground glass opacities or crazy‐paving appearance. These findings suggested that fibrotic progression was the major process, although recurrence or residual activity of APAP could not be completely excluded on radiological grounds alone. No clinical features suggestive of connective tissue disease or clear exposure to inhalational antigens were identified. Serological tests, including antinuclear antibodies, rheumatoid factor, and myeloperoxidase antineutrophil cytoplasmic antibody, were negative. Initial pulmonary function tests showed a FVC of 3.31 L (80.0% predicted), indicating mildly reduced lung volume. The diffusion capacity for carbon monoxide (DL_CO_) was 23.63 mL/min/mmHg (103.1% predicted), indicating preserved gas transfer. Pulmonary function tests had not been performed at the previous hospital; therefore, whether the patient fulfilled the diagnostic criteria for PPF before referral was unclear.

**Figure 2:**
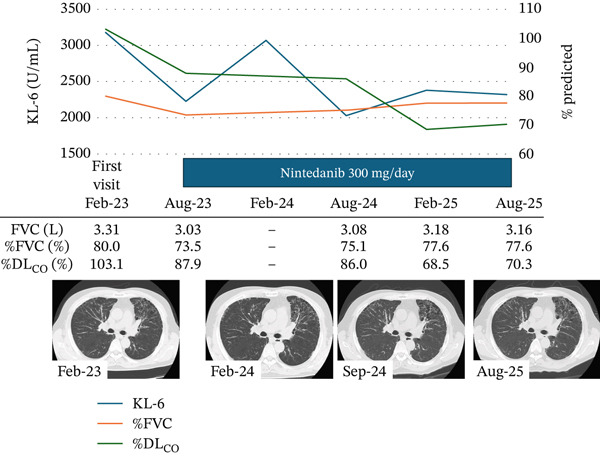
Clinical course after referral to our hospital. During a careful observation period of 6 months after the first visit, the patient′s forced vital capacity (FVC) decreased by 280 mL, corresponding to an absolute decrease of 6.5% in percent predicted FVC (%FVC). Following the initiation of nintedanib, FVC remained relatively stable, exertional dyspnea did not worsen, and follow‐up chest CT showed no clear radiological progression of fibrosis. In contrast, DL_CO_ decreased from 87.9% at the start of nintedanib to 70.3% at the final follow‐up, indicating that the physiological course was not uniformly stable. Missing pulmonary function data are indicated by a dash. DL_CO_, diffusion capacity of the lung for carbon monoxide; KL‐6, Krebs von den Lungen‐6.

Based on his medical history, radiological findings, and pulmonary function, we opted for close observation. Six months later, serial pulmonary function tests showed that FVC had decreased to 3.03 L (73.5% predicted). This decline was accompanied by worsening dry cough and dyspnea on exertion. The patient was clinically considered to have developed a progressive fibrosing phenotype in the context of long‐standing APAP. This assessment was based primarily on worsening of exertional dyspnea within the year before referral and an absolute decline in FVC of 6.5% over a 6‐month observation period after referral, corresponding to the symptom and physiological domains of the 2022 ATS/ERS/JRS/ALAT PPF criteria [2]. Although radiological progression within the same standardized 1‐year interval could not be confirmed, long‐term serial HRCT images showed gradual progression of reticulation and traction bronchiectasis, supporting the fibrosing nature of the disease. Based on this clinical assessment, nintedanib was initiated at a dose of 300 mg/day. Over the subsequent 2 years, FVC remained relatively stable without further marked decline. Exertional dyspnea improved after treatment initiation and did not worsen during follow‐up. Follow‐up chest CT showed no clear radiological progression of fibrosis. In contrast, DL_CO_ gradually decreased from 87.9% predicted at the start of nintedanib to 70.3% predicted at the final follow‐up (Figure 2). Therefore, although there was no clear symptomatic or radiological progression after nintedanib initiation, the physiological course was not completely stable, and careful follow‐up was continued. Systematic evaluation for pulmonary hypertension, such as transthoracic echocardiography or right heart catheterization, was not performed during follow‐up.

## 3. Discussion

This report describes a patient with long‐standing APAP who developed fibrosing lung disease with features consistent with a progressive fibrosing phenotype and was treated with nintedanib during the clinical course. This case highlights the difficulty of distinguishing fibrotic progression from recurrence of intra‐alveolar proteinosis and of interpreting physiological changes after antifibrotic therapy in patients with APAP.

Pulmonary fibrosis has been reported in patients with APAP. Ono et al. [5] reported on a patient with APAP‐associated progressive fibrosis who underwent repeated WLL and inhaled GM‐CSF therapy. Luisetti et al. [6] reported cases suggesting a possible relationship between diffuse pulmonary fibrosis, alveolar proteinosis, and anti‐GM‐CSF autoantibodies. More recently, Guirriec et al. [7] found that 16 of 61 patients with APAP (26%) had pulmonary fibrosis in a nationwide retrospective cohort. In that study, fibrotic abnormalities were mainly identified by CT findings such as traction bronchiectasis or bronchiolectasis, and patients with fibrosis had worse clinical outcomes than those without fibrosis. These findings suggest that fibrotic lung disease is a clinically significant complication in some patients with APAP. However, the disease behavior of APAP‐associated fibrosis, including its overlap with the current multidimensional concept of PPF, remains insufficiently defined.

The pathophysiology of fibrosing lung disease in APAP may differ from that of IPF. In APAP, anti‐GM‐CSF antibodies impair alveolar macrophage function and surfactant clearance, and persistent intra‐alveolar surfactant accumulation may promote chronic alveolar epithelial injury, abnormal repair, and fibrotic remodeling. In contrast, IPF is generally regarded as a fibrosing interstitial pneumonia driven by repetitive alveolar epithelial injury and aberrant repair without an identifiable underlying cause. PPF is a clinical behavior concept rather than a specific etiology. Therefore, APAP may have provided a disease‐specific background for fibrotic remodeling in this patient, although the exact mechanism could not be determined without histopathological confirmation.

A key issue in the present case was the distinction between recurrence of APAP and progression of fibrosing lung disease. The typical radiological manifestations of APAP are ground‐glass opacities and a crazy‐paving appearance. These manifestations reflect the accumulation of surfactant‐derived material in the alveoli. In contrast, our patient showed a gradual progression of reticulation and traction bronchiectasis without clear radiological evidence of APAP recurrence. There were no clinical features suggestive of connective tissue disease, nor was there clear exposure to inhalational antigens. Serological tests for connective tissue disease–related interstitial lung disease were negative. Taken together, these findings supported the clinical interpretation that fibrotic progression was the predominant disease process in the context of long‐standing APAP, rather than recurrence of APAP or another identifiable fibrosing interstitial lung disease. However, we acknowledge that recurrence or residual activity of APAP cannot be completely excluded solely on radiological grounds, particularly because repeat bronchoalveolar lavage and anti‐GM‐CSF antibody testing were not performed at referral. Thus, the radiological findings should be interpreted as supporting predominant fibrotic progression, not as definitively excluding APAP‐related activity.

The serum KL‐6 trend required careful interpretation. Although KL‐6 is a useful biomarker in interstitial lung diseases, it is often markedly elevated in PAP and may reflect intra‐alveolar surfactant accumulation and epithelial injury. In this case, KL‐6 decreased initially and then fluctuated while remaining elevated, possibly reflecting partial attenuation of APAP activity rather than improvement of fibrosis. Persistent elevation did not exclude fibrotic abnormalities, as KL‐6 alone cannot distinguish APAP activity from fibrosing lung disease. Therefore, KL‐6 was not used to define the progressive fibrosing phenotype; assessment was based on radiological progression, symptom worsening, and FVC decline.

In this case, the diagnosis of a progressive fibrosing phenotype requires careful but not overly restrictive interpretation. The patient had worsening exertional dyspnea within the year before referral and showed an absolute decline in %FVC of 6.5 percentage points over 6 months after referral, corresponding to the symptom and physiological domains of the 2022 ATS/ERS/JRS/ALAT PPF criteria [2]. Radiological progression within the same standardized 1‐year interval could not be confirmed. However, long‐term serial HRCT images showed gradual progression of reticulation and traction bronchiectasis, supporting the fibrosing nature of the disease. Thus, the decision to initiate nintedanib was based mainly on symptom worsening and physiological progression, with long‐term radiological progression providing supportive evidence that the underlying disease process was fibrosing lung disease.

The role of antifibrotic therapy in APAP‐associated fibrosing lung disease remains unclear. Nintedanib has been shown to reduce FVC decline in patients with progressive fibrosing interstitial lung diseases [3], but patients with APAP‐associated fibrosing lung disease are likely to be underrepresented or absent from major clinical trials. A recently published report described a patient with PAP‐associated interstitial lung disease treated with nintedanib without success [8]. However, that case showed a cellular non‐specific interstitial pneumonia pattern rather than a chronic progressive fibrosing phenotype. In the present case, FVC remained relatively stable for 2 years after the initiation of nintedanib, and respiratory symptoms and follow‐up CT findings did not show clear worsening. In contrast, DL_CO_ declined from 87.9% predicted at the start of nintedanib to 70.3% predicted at the final follow‐up. This decline was clinically meaningful and indicates that the physiological course was not uniformly stable. Therefore, stabilization of FVC alone is insufficient to establish treatment efficacy.

The discrepancy between relatively stable FVC and declining DL_CO_ requires careful interpretation. FVC mainly reflects lung volume and restrictive impairment, whereas DL_CO_ may be affected by gas‐exchange abnormalities at the alveolar‐capillary interface, residual or fluctuating APAP‐related intra‐alveolar disease activity, and pulmonary vascular disease. Thus, treatment response in APAP‐associated fibrosing lung disease should be evaluated using multiple domains, including symptoms, FVC, DL_CO_, and radiological findings. This case suggests that antifibrotic therapy may be a therapeutic option for selected patients with fibrosing lung disease in the context of APAP, whereas careful multidimensional assessment remains essential.

Assessment of APAP activity is important when determining management. DSS‐based evaluation, including symptoms and oxygenation status, is useful for assessing APAP severity and for guiding APAP‐directed treatments such as WLL or GM‐CSF therapy [9]. In the present case, PaO_2_ was 68 Torr in room air at referral, corresponding to DSS grade 3. However, nintedanib was initiated not for active intra‐alveolar proteinosis itself, but for fibrosing lung disease with progressive fibrosing behavior, based on worsening symptoms, FVC decline, and fibrotic abnormalities on HRCT. If APAP‐associated fibrosis progresses to advanced respiratory failure, lung transplantation may become a therapeutic option. Corticosteroid therapy was not used in this case because corticosteroids are generally unfavorable in PAP [10].

This case has several limitations. Bronchoscopic or histopathological confirmation of the fibrosing process was not available, and longitudinal pulmonary function data before referral were unavailable. In addition, anti‐GM‐CSF antibody was not reassessed at the time of referral; therefore, persistent antibody positivity could not be confirmed, and a direct causal relationship between APAP and the subsequent fibrosing lung disease cannot be proven. In addition, recurrence or residual activity of intra‐alveolar proteinosis could not be completely excluded because bronchoalveolar lavage was not repeated and the interpretation was based mainly on clinical and radiological findings. Although nintedanib was associated with relative stabilization of FVC, symptoms, and radiological findings, DL_CO_ declined during follow‐up. Systematic evaluation for pulmonary hypertension was not performed, and pulmonary vascular disease could not be excluded as a contributor to the decline in DL_CO_. Therefore, the clinical course of a single case cannot establish a treatment effect. These limitations preclude firm conclusions regarding disease classification and the efficacy of antifibrotic therapy.

In conclusion, APAP‐associated fibrosing lung disease may present with progressive fibrosing behavior. This case highlights the importance of distinguishing fibrotic progression from recurrence of intra‐alveolar proteinosis and of carefully evaluating physiological changes after antifibrotic therapy. Further case accumulation is needed to clarify the clinical course and management of APAP‐associated fibrosing lung disease.

**Nomenclature**
APAP: autoimmune pulmonary alveolar proteinosis
DL_CO_
: diffusion capacity for carbon monoxide
FVC: forced vital capacity
GM‐CSF: granulocyte‐macrophage colony‐stimulating factor
HRCT: high‐resolution computed tomography
PAP: pulmonary alveolar proteinosis
PPF: progressive pulmonary fibrosis
WLL: whole‐lung lavage

## Author Contributions

Y.Z. conceptualized and drafted the manuscript. Y.N., T.J., T.K., and K.I. critically reviewed and edited the manuscript. T.T. and T.H. organized and contributed to the management of this case report.

## Funding

No funding was received for this manuscript.

## Disclosure

All authors read and approved the final version of the manuscript.

## Ethics Statement

Ethics approval was not required for this case report in accordance with institutional policy. Written informed consent was obtained from the patient.

## Consent

Written informed consent for publication of this case report and any accompanying images was obtained from the patient.

## Conflicts of Interest

Y.Z. and T.T. received lecture fees from Nippon Boehringer Ingelheim Co., Ltd. T.T. also received lecture fees from AstraZeneca K.K. The other authors declare no conflicts of interest.

## Data Availability

Data is sharing not applicable to this article as no datasets were generated or analyzed during the current study.
